# Droplets microfluidics platform—A tool for single cell research

**DOI:** 10.3389/fbioe.2023.1121870

**Published:** 2023-04-19

**Authors:** Bixuan Li, Xi Ma, Jianghong Cheng, Tian Tian, Jiao Guo, Yang Wang, Long Pang

**Affiliations:** ^1^ Xi’an Key Laboratory of Pathogenic Microorganism and Tumor Immunity, Xi’an, China; ^2^ School of Basic Medicine, Xi’an Medical University, Xi’an, China

**Keywords:** single-cell culture, tumor single-cell immunity, droplets microfluidics, single cell detection, single cell screening

## Abstract

Cells are the most basic structural and functional units of living organisms. Studies of cell growth, differentiation, apoptosis, and cell-cell interactions can help scientists understand the mysteries of living systems. However, there is considerable heterogeneity among cells. Great differences between individuals can be found even within the same cell cluster. Cell heterogeneity can only be clearly expressed and distinguished at the level of single cells. The development of droplet microfluidics technology opens up a new chapter for single-cell analysis. Microfluidic chips can produce many nanoscale monodisperse droplets, which can be used as small isolated micro-laboratories for various high-throughput, precise single-cell analyses. Moreover, gel droplets with good biocompatibility can be used in single-cell cultures and coupled with biomolecules for various downstream analyses of cellular metabolites. The droplets are also maneuverable; through physical and chemical forces, droplets can be divided, fused, and sorted to realize single-cell screening and other related studies. This review describes the channel design, droplet generation, and control technology of droplet microfluidics and gives a detailed overview of the application of droplet microfluidics in single-cell culture, single-cell screening, single-cell detection, and other aspects. Moreover, we provide a recent review of the application of droplet microfluidics in tumor single-cell immunoassays, describe in detail the advantages of microfluidics in tumor research, and predict the development of droplet microfluidics at the single-cell level.

## 1 Introduction

The cell is the basic functional unit of all organisms. Microbial cells can together form a large biochemical network of complex, coordinated chemical reactions. Microbial cells in a group interact methodically and can catalyze the production of vital compounds that are difficult to chemically synthesize *in vitro* (Schmid et al., 2001), thus ensuring the normal operation of various functions of the human body and performing various life movements. Understanding cells’ morphology, composition, function, and genetic properties are of great significance for understanding biological principles, mapping the human genome, and diagnosing and treating diseases. However, most cell studies have historically been limited to the overall macroscale analysis of cell populations (Fritzsch et al., 2012). In recent years, with the development and application of bioengineering technologies, such as flow cytometry and microfluidic technology, it has become possible to assess single cells and thus observe the high level of heterogeneity between cells. There are great differences between single cells in terms of morphology, size, gene expression, and growth characteristics (Bowes et al., 2022; Wang et al., 2022). Even single cells from the same population demonstrate many examples of heterogeneity in culture (Colman-Lerner et al., 2005; Kaern et al., 2005; Newman et al., 2006). Therefore, analysis at the single-cell level becomes particularly important: single-cell culture reveals cells’ morphological and functional heterogeneity in a controlled environment and enables monitoring of physiological changes during cell growth; single-cell screening technology further helps address the difficulties caused by cell heterogeneity in the diagnosis of diseases (Kothalawala et al., 2022; Pearson et al., 2022). According to differences in cell morphology or secretion in response to external stimuli, rapid, high-throughput screening of the desired target cells makes it possible to find rare cells. Single cells contain biomolecules, such as DNA, RNA, protein, or other cell surface molecules. The sensitive and accurate measurement of small molecules at the single-cell level is crucial for studying cell genomics, proteomics, and biochemical mechanisms.

In recent years, researchers have focused on developing new single-cell techniques. Flow cytometry and microfluidic techniques stand out as the most effective tools for single-cell analysis. Flow cytometry has the advantage of high throughput and can detect and analyze tens of thousands of individual cells quickly. It is commonly used for protein expression analysis of cell populations. However, tracking of specific single cells, culturing of single cells, and quantification of single-cell products cannot be achieved in flow cytometry. Microfluidic technology has emerged as a solution to these problems. Due to its flexible manipulability, low volumes, and high flux, microfluidic technology has become a popular platform for single-cell research in recent years (Suea-Ngam et al., 2019; Li et al., 2021).

**FIGURE 1:**
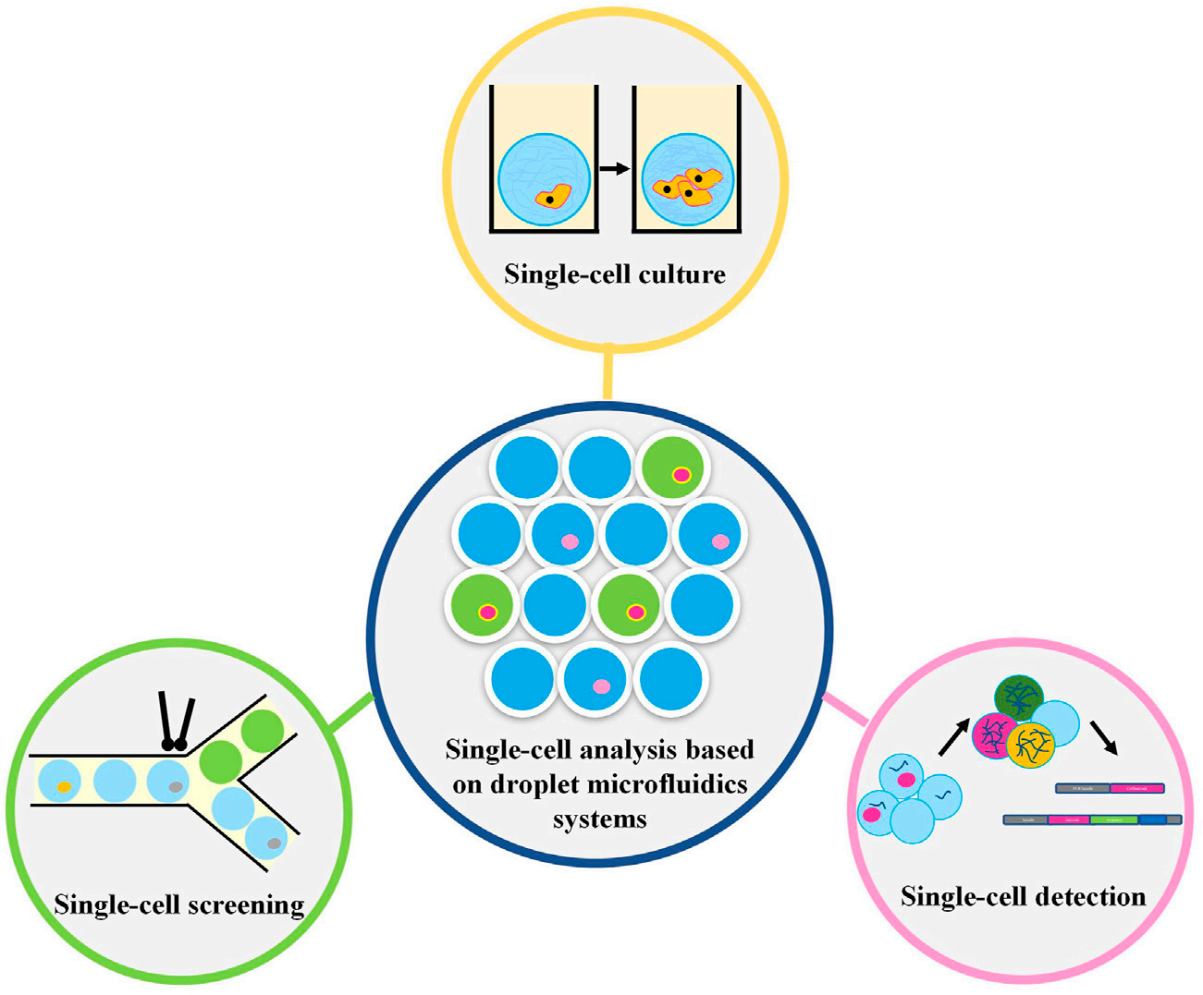
The single-cell analysis based on droplet microfluidics systems.

Most of the previous reviews focused on single cell droplet generation and the application of droplet microfluidics in one aspect of single cell research, such as single cell omics, pathogen detection, drug screening, etc. Different from previous reviews. This paper systematically introduces droplet generation, single cell encapsulation, single cell culture and single cell technology based on droplet microfluidics, aiming to provide ideas for researchers who first contact droplet microfluidics single cell analysis (Figure 1). In particular, we introduced the application of droplet microfluidics in tumor cell analysis, and discussed the existing problems, challenges and application prospects of droplet microfluidic in tumor immunity and tumor therapy, aiming to provide new ideas for the development of tumor immune detection platform.

## 2 Droplet generation and single-cell encapsulation

Microfluidics involves the processing and manipulating 10^−8^–10^−9^ L of fluid through pumps or valves in a chip channel that is tens to hundreds of microns. With the continuous progress of micromachining technology, channel design is becoming increasingly precise, which can facilitate the integrated automation of the research and analysis of complex fluids. Therefore, such a microfluidic chip is also known as a “micro total analysis system (µTAS)” or “lab-on-a-chip (Manz et al., 1990; Xue et al., 1997; Dittrich et al., 2006).” Droplet microfluidics is a very important branch of microfluidic technology in which two immiscible fluids are placed into predesigned microfluidic channels, and the flow rate is controlled to cut the two phases at the interface, a process that can thus form tens of thousands of homogeneous droplets. The droplet generation rate and size can be controlled by the two-phase flow rate, and the smallest droplets can be on the micron or even nanometer scale. Each droplet acts as a separate reactor isolated from the outside world. Therefore, droplet microfluidics, as a high-throughput screening platform with great potential, has been widely used in enzyme reaction kinetics, cell culture, nucleic acid detection and analysis, protein crystallization, and many other fields (Ou et al., 2021; Liu et al., 2022; Postek and Garstecki, 2022; Jiang et al., 2023). This chapter mainly introduces droplet generation technology and how to prepare emulsions that encapsulate single cells, providing basic ideas and methods for researchers beginning to work with droplet microfluidics*.*

### 2.1 Generating droplets

**FIGURE 2:**
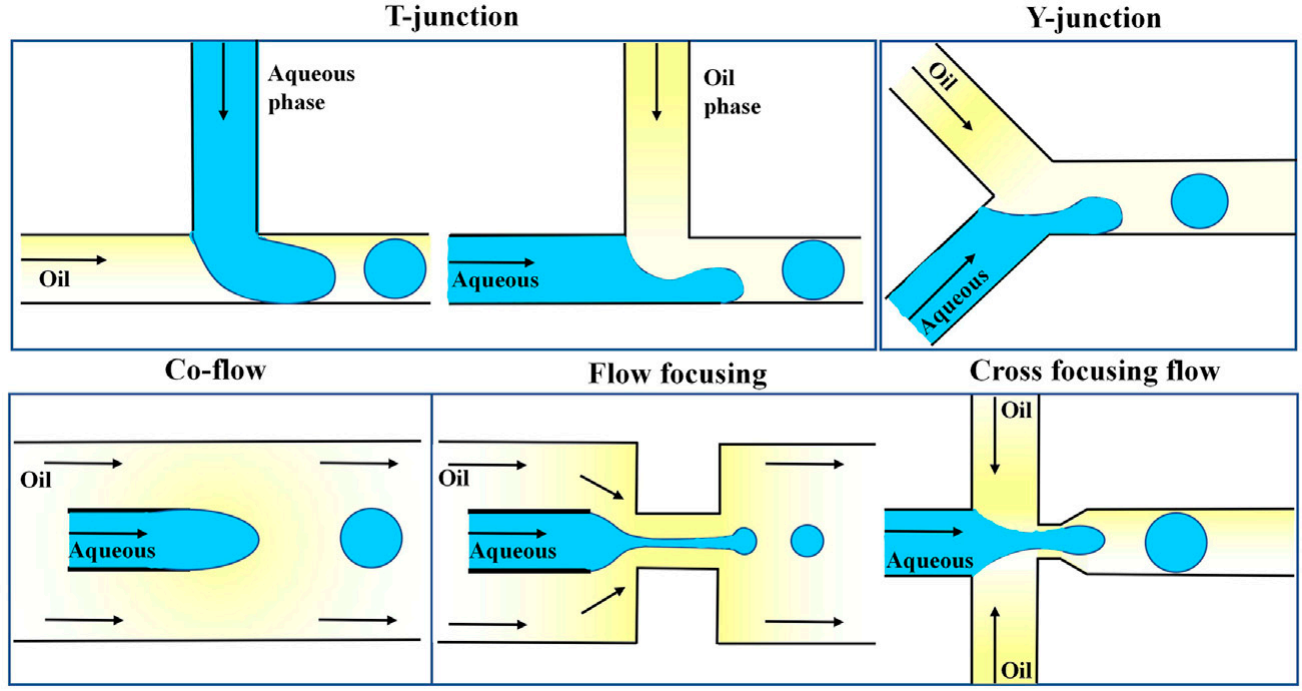
Different channel designs for droplet generation (T, Y, co-flow, flow focusing, and cross-focusing flow) (Vladisavljevic et al., 2013).

Droplets can be generated by using two immiscible liquids, one of which is separated into the other by shear forces. Commonly used channel designs include T-type, X-type, Y-type, co-flow-type, and focused flow-type designs (Stone et al., 2004; Vladisavljevic et al., 2013), as shown in Figure 2. The T-channel is the simplest channel design for droplet generation (Yi et al., 2003; Van der Graaf et al., 2005). The oil phase (continuous phase) and water phase (dispersed phase) meet at an inlet.

Due to the two liquids’ immiscibility and the channel junction’s design, one liquid is shaped into droplets within the other liquid. The oil is connected to the main channel in the standard geometry, and the water phase is injected through an orthogonal channel. Under the combined effect of the oil phase’s shear force and the water phase’s upstream pressure, the aqueous phase’s downstream part is squeezed and eventually cut off into small droplets (Garstecki et al., 2006; Zhao and Middelberg, 2011). Xu et al. optimized such a system by altering the shapes of the water and oil channels, and thus the oil phase in the intersecting channel directly cut the water phase under pressure (Xu et al., 2006). The size of droplets in t-shaped channels can be controlled by changing the two-phase flow rate by, for example, externally controllable push pumps, integrated microheaters, and pneumatic or magnetically driven microvalves (Lin and Su, 2008; Lee et al., 2009; Murshed et al., 2009; Yamanishi et al., 2010). The droplet generation principle of the Y-type channel is similar to that of the T-type channel (Steegmans et al., 2009). The common design generally consists of two channels arranged coaxially, and the inner channel is often designed with a tapered tip (Umbanhowar et al., 2000). Droplets are generated from the inner tube, and when the viscous resistance exerted by the oil phase exceeds the interfacial tension, the water phase is cut off to form droplets. A focused flow channel forces a two-phase liquid through a small hole to generate droplets (Xu and Nakajima, 2004). Anna and Mayer (2006) added a very narrow channel into the common flow channel, and the oil and water phases entered the small tube together in two directions. Compared with the co-flow, the pressure and shear force exerted by the continuous relative dispersed phase were enhanced, which was more conducive to the formation of droplets. The scientists went a step further and invented a cross-focusing flow channel, in which the oil phase passes through two longitudinal channels, and the water phase is cut at the junction of the two phases. The cross-focusing flow microfluidic channel is a droplet generation channel commonly used in recent years.

The droplets generated by the channels need to be collected and stored for subsequent analysis. However, the microdroplet emulsion prepared by the channel is in a metastable state without surfactant stability, and droplets fuse upon contact after collection (Theberge et al., 2010). Surfactants are stable in a particular two-phase solution and can maintain droplets for hours or even years. A review by Baret provides an in-depth look at the role of surfactants in droplet generation chips (Baret, 2012). Common oil phases used oil-in-water emulsions are hydrocarbon oil and fluorocarbon oil, which are stabilized by commercially available surfactants, typical examples being Span80, Arbil EM, and PFPE (Link et al., 2004; Courtois et al., 2008; Holtze et al., 2008). Compared with hydrocarbon oil, surfactant-modified fluorinated oil has better oxygen transport performance and can not only prolong droplet retention time but also has been demonstrated to have high biocompatibility (Clausell-Tormos et al., 2008).

With the rapid development of technology and Internet, droplet generation microfluidic devices have made new breakthroughs. Recently, Lashkaripour et al. (2021) present a web-based tool, DAFD (Design Automation of Fluid Dynamics), that predicts the performance and enables design automation of flow-focusing droplet generators. They capitalize on machine learning algorithms to predict the droplet diameter and rate with a mean absolute error of less than 10 μm and 20 Hz. They demonstrate that DAFD can be extended by the community to support additional fluid combinations, without requiring extensive machine learning knowledge or large-scale data-sets. This tool will reduce the need for microfluidic expertise and design iterations and facilitate adoption of microfluidics in life sciences.

### 2.2 Single-cell encapsulation

In the past few decades, droplet microfluidics technology has developed rapidly, and it has been used in the design of microreactors because of its strong controllability in performing various operations (Chen et al., 2022; Jobdeedamrong et al., 2023). Due to their compatibility with many chemical and biological reagents, droplet microfluidic systems have been used to prepare complex reactants and special materials, such as polymer particles (Juang and Chiu, 2022), microgel particles (Rojek et al., 2022), and nanoparticles (Küçüktürkmen et al., 2022; Maged et al., 2022; Niculescu et al., 2022). Moreover, droplet microfluidics is widely used in biomedical research due to their simple and easy operation and high-throughput encapsulation capability. For example, droplet microfluidics has been used in drug encapsulation (Li et al., 2022; Chen et al., 2023), targeted drug delivery (Jia et al., 2022; Sartipzadeh et al., 2022), and multi-functionalization of carriers (Wu et al., 2010).

Ideally, each droplet used for single-cell analysis should contain only one cell since the number of cells per droplet significantly affects downstream research analysis. However, because cells suspended in the dispersed phase arrive at the oil-water interface randomly, the number of cells encapsulated in the droplet cannot be predicted (JYu et al., 2014; David et al., 2015). Scientists have estimated the fraction of droplets containing cells when passively encapsulated from a Poisson distribution Eq. 1.

$$p\left(k,\lambda \right)=\frac{\lambda^{k}e^{-\lambda }}{k!}$$ (1)

where k is the number of cells encapsulated in each droplet and *λ* is the average number of cells in each droplet. Calculations show that when *λ* = 1, only approximately 58% of the resulting droplets contain cells, and only 36% contain only one cell. Due to errors in the actual experiment, these numbers often cannot be met, and the experiment falls far below the basic requirements of single-cell analysis. Therefore, to improve encapsulation efficiency, scholars have developed many methods of passive and active cell encapsulation (Carlo et al., 2007).

**FIGURE 3:**
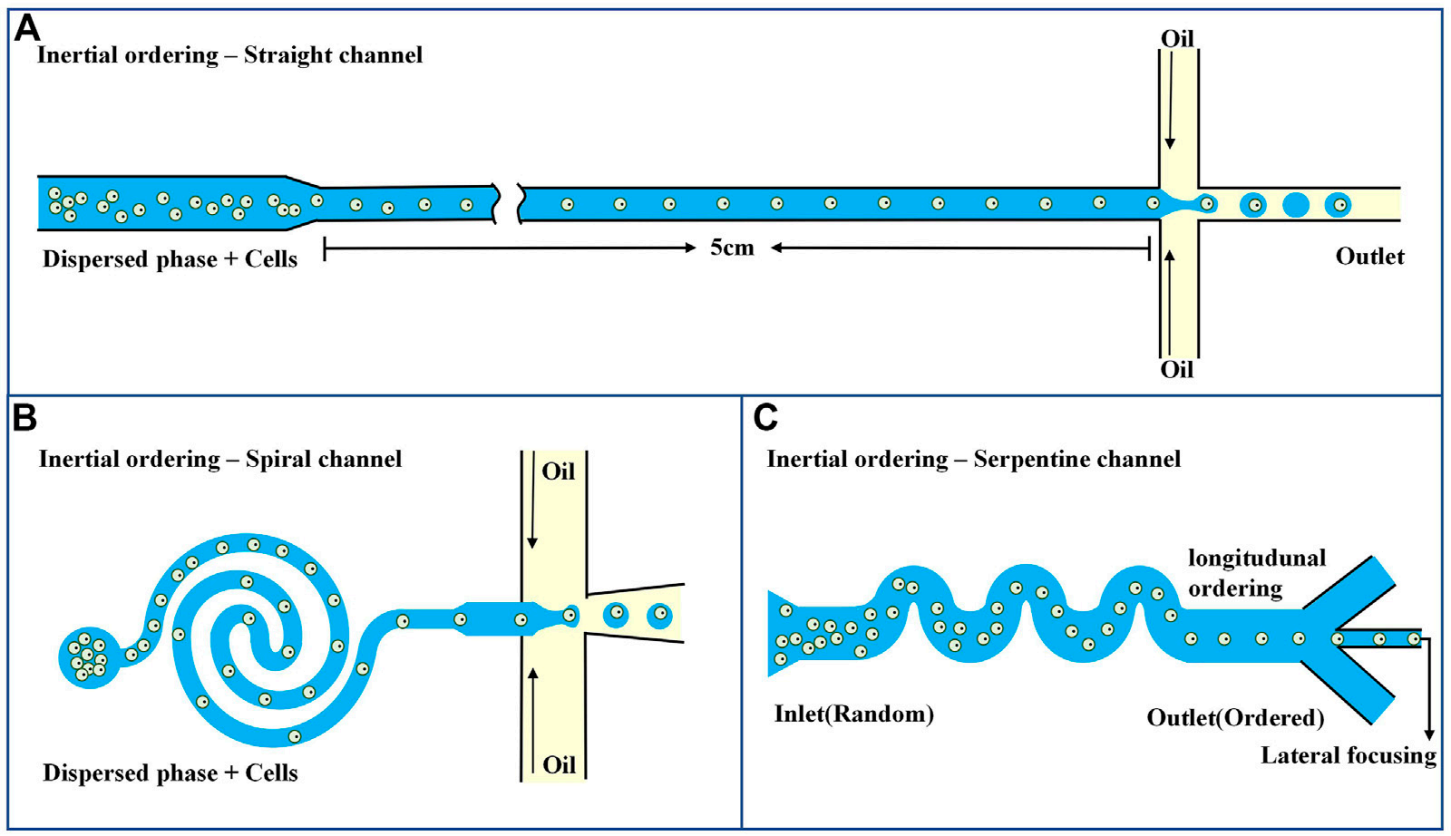
Microfluidics-based systems for single-cell focusing and encapsulation **(A)** Passive encapsulation of straight channel (Ling et al., 2020). **(B)** Passive encapsulation of spiral micro channel (Ling et al., 2020). **(C)** Passive encapsulation of serpentine channel (Li et al., 2019).

The common method of passive encapsulation is to transport the suspended cells via a microchannel and then arrange and deliver them to the droplet encapsulation area. The distance between neighboring suspended cells can be controlled by changing the cell concentration, input flow, or microchannel size. As shown in Figures 3A, B, linear and spiral microchannels are commonly used. Regardless of which one is used, hydrodynamic balance in the microchannels is required to achieve the inertial and orderly flow of suspended cells. In addition, the channels need to be long enough to ensure sufficient time to sequence the cells before encapsulation, and it has been reported that the straight and spiral channels are generally 5 cm and 1 cm long, respectively (Ling et al., 2020). To avoid excessively long channels, Li et al. (2019) added curved serpentine channels after spiral channels to shorten the time for cells to reach the specified area (Figure 3C). Active cell encapsulation increases the number of droplets containing only one cell by manipulating cell displacement or adjusting the rate of droplet production. A detection module, such as a fluorescence detection section, is integrated into the microfluidic system to predict the arrival of individual cells. Once the target cell reaches the junction formed by the droplet, an external force is applied to actively trigger the formation of the droplet containing the single cell. Compared with simple passive encapsulation, it can shorten encapsulation time and improve encapsulation efficiency. Commonly used external forces include electric and magnetic forces (Hao et al., 2011; Chen et al., 2013).

## 3 Single-cell analysis technique based on droplet microfluidics

Biological systems have evolved from single cells, and even single cells in the same population are highly heterogeneous; for example, bacterial mutations can lead to antibiotic resistance, and changes in tumor cell genomes enable the tumor cells to evade immune responses (Ghosh and Saha, 2020; Peña-Romero and Orenes-Piñero, 2022). Therefore, studies at the single-cell level are necessary to understand a cell population. A single-cell culture is a basic tool for single-cell research. Cultivating and understanding single-cell behavior for biological and clinical research is very important. The development of microfluidic technology has facilitated high-throughput single-cell cultures. Each droplet acts like a small cell culture chamber. Adjusting the continuous and dispersed phases can ensure that the nutrient environment and material exchange with the outside world needed for cell growth can be satisfied in the droplet (Zhu and Yang, 2016; Huang et al., 2017). Droplet microfluidics allows for the precise control of the microenvironment of a single cell culture, manipulation of a single cell through external intervention, and real-time detection of single-cell behavior for enzyme, antibody, or rare cell screening. Even after culture, cells can be recovered from the microfluidic system for various downstream analyses. The excellent specific surface area of the droplet allows it to transfer heat rapidly and uniformly for high-speed reactions (Kaminski and Garstecki, 2017). In addition, the microfluidic chip can facilitate cell separation, culture, analysis, and detection. Thus, the multiscale multifunctional operation can be achieved on a small chip, and data can be obtained at the single-cell level that cannot be obtained from the culture of entire cell populations.

### 3.1 Single-cell culture

*In vitro* cell culture, especially mammalian cell culture requires a culture dish or flask to provide nutrients for cell growth in a proper gas atmosphere, and cell concentrations need to be controlled such that the cultures do not exceed growth limits and are not affected by apoptotic cells (Eibl et al., 2018; Jensen and Teng, 2020; Badr-Eldin et al., 2022). In droplet microfluidics chips, cell culture is divided into droplet compartments, and culture medium and growth factors are added to the continuous phase to provide the nutrients needed for cell growth. While the droplets insulate cells from contamination, perfluorocarbon oils provide air permeability and allow the cells to exchange air with the outside world. Bacteria, yeast, and mammalian cells (Huebner et al., 2007; Köster et al., 2008; Pang et al., 2021; Seiler et al., 2022) have been proven able to survive encapsulated in droplets for a long time and retain good cell activity after resuscitation, which can be used for various biological measurements. Compared with traditional culture methods, fewer reagents are consumed, but vitality is slightly decreased (Nakagawa et al., 2021). In one study, cells were suspended in a culture medium, and droplets were prepared according to a Poisson distribution (Staszewski, 1984; Moon et al., 2011); after many dilutions, single-droplet single-cell encapsulation was achieved, but up to 90% of the droplets were empty (without cells). Various methods have been developed to overcome this problem, such as encapsulation by the self-organizing process of cells (Edd et al., 2008) or separation of droplet-containing cells from empty droplets according to Rayleigh Plateau instability (Chabert and Viovy, 2008).

**FIGURE 4:**
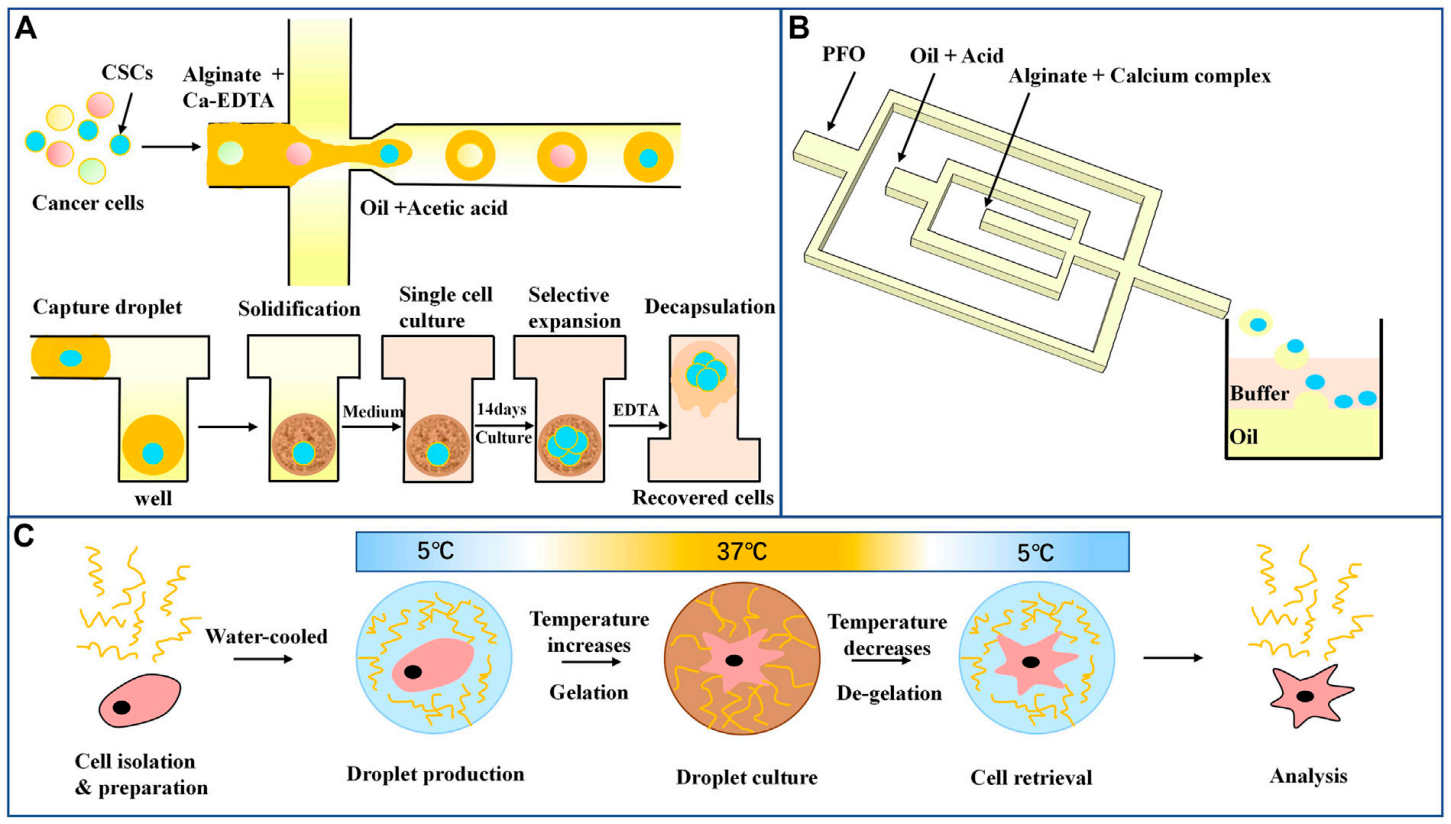
Droplet microfluidics for single-cell culture **(A)** Schematic diagram of sodium alginate droplet arrays for cancer stem cell culture and screening (Lin et al., 2021). **(B)** Microfluidic encapsulation of single cells by alginate microgels using a trigger-gelation strategy. Nitrilotriacetic acid (NTA) complex alginate drops were demulsified in a neutral pH environment (Shao et al., 2020). **(C)** In the hydrogel and cell encapsulation workflow, cells (red) are encapsulated with hydrogel (blue) at 5°C; as temperature increases in the cell incubator, gelation occurs, and after the desired culture period, droplets are cooled followed by de-emulsification, resulting in easy cell retrieval (Tiemeijer et al., 2021).

Hydrogel droplets can be rapidly generated by microfluidic devices using a monomer or polymer solution as the water phase. Compared with ordinary water droplets, hydrogel droplets have advantages such as natural biocompatibility and phase transformation in response to external physical and chemical changes, and they have been widely used in single-cell cultures. Sodium alginate hydrogel is the most commonly used natural hydrogel, and it can be cured by reaction with Ca^2+^ (Moyer et al., 2010). To produce droplets from microfluidics that rapidly solidified, the researchers allowed for the diffusion of the granular crosslinker from the continuous phase into the dispersed phase or made droplets in a high-concentration Ca^2+^ environment (Huang et al., 2006; Tan and Takeuchi, 2007). However, these methods cause many problems, such as uneven hydrogel formation, the formation of large microspheres, and high cytotoxicity due to long-term leakage of surfactant and high cross-linking agent concentrations (Zhang et al., 2007; Choi et al., 2016). To overcome these difficulties, Utech and others used soluble ethylenediamine tetraacetic acid (EDTA)-Ca^2+^ complex compound as the cross-linking agent of alginate (Utech et al., 2015). Under acidic conditions, Ca^2+^ can be replaced by sodium alginate for curing, and rejoining the EDTA complexometric to dissolve Ca^2+^ can cure the microspheres. Lin (Lin et al., 2021) designed a microfluidic array for culture enrichment and screening of colon cancer stem cells using sodium alginate droplets, as shown inFigure 4A. The alginate droplets that encase the cancer cells are captured in the array, calcified, and solidified under acidic conditions. The oil-phase infusion medium was removed, and the cells were cultured in a single-cell environment for 14 days. EDTA-melted microspheres were added again to collect cells for subsequent analysis. Only tumor stem cells can expand and form cell spheres without adhesion, while normal cancer cells gradually die due to the loss of adhesion and starvation. By comparing the transcriptome of cells collected by single-cell culture with that of adherent culture cells, it was found that individually cultured cells had a series of genes that were significantly upregulated and had a higher ability to maintain stem cell characteristics, multiline differentiation, and stress resistance. However, due to the influence of an acidic environment, there was some loss in the cell recovery process. Shao et al. (2020) used nitrilotriacetic acid (NTA)-Ca^2+^ complexes to promote sodium alginate calcium. As shown in Figure 4B, the inner cross-focusing flow channel generates sodium alginate droplets in solidified oil. In contrast, the outer channel passes through perfluorooctanoate (PFO) to disrupt the stability of the oil-water interface. The microgel transfers from the oil to the water phase composed of the buffer until the pH of the microgel is neutralized. Compared with EDTA, the NTA complex has a higher dissociation constant due to the release of Ca^2+^ in a neutral environment. The encapsulated cells also showed higher activity and cell metabolic activity, and the researchers also used an osteogenetic differentiation model to show the long-term function of mesenchymal stem cells. This biocompatible strategy of cell encapsulation using microfluidic chips provides a powerful tool for future applications of alginate microgels in tissue engineering and cell therapy.

An important function of single-cell culture is to analyze the heterogeneity of cells and elucidate the cell uniqueness obscured by the homogenization of cell populations. However, sodium alginate gel curing requires the action of a cross-linking agent, some of which affects cell biological activity. Some natural hydrogels, such as agarose and collagen (Leng et al., 2010; Dolega et al., 2015), can undergo phase transformation in response to temperature. However, agarose and collagen gel require complex temperature manipulation or the addition of reagents to ensure droplet formation and cell culture. Polyisocyanate (PIC) hydrogels are a promising alternative with a gel temperature of 15°C that allows droplet preparation at lower temperatures and hydrogel formation at cell culture temperatures. In addition, by cooling, the gel quickly reverses, facilitating cell recovery. Tiemeijer et al. (2021) combined a microfluidic droplet platform with a thermally reversible PIC hydrogel to study the heterogeneity of highly adherent human macrophages. As shown in Figure 4C, single macrophages were isolated and cultured based on the droplet microfluidics method and then stimulated. Monocytes were first isolated from healthy human blood and differentiated into macrophages. The macrophages and stimulating factors were mixed on ice to ensure no premature cellular activation. The cells were then encapsulated in microliter droplets and incubated for a while. After breaking the emulsion droplets, the cells were extracted by PFO, and phenotypic analysis was performed by flow cytometry. It was found that encapsulation of multiple cells enhanced cell polarization compared with a single cell, indicating that cell communication was a strong driver of macrophage polarization. Furthermore, the authors found that single macrophages cultured in PIC hydrogel drops showed higher cell viability and enhanced M2 polarization than single macrophages cultured in suspension culture. Notably, combined with phenotypic and functional analysis of individually cultured macrophages, a subpopulation of cells in a persistent M1 state was found, which was not detectable in traditional cultures. In conclusion, combining droplet microfluidics with hydrogels is a versatile and powerful tool for studying the biological functions of adherent cell types at single-cell high flux resolution. Single-cell culture and single-cell function analysis are excellent techniques for discovering new populations of immune cells.

### 3.2 Single-cell screening

Single-cell studies can not only reflect the heterogeneity among individual cells but also analyze the complex responses of cells to various physiological and pathological stimuli at the single-cell level. Identifying rare cells is important for diagnosing and prognosis of diseases (Saadatpour et al., 2015; Ziegler et al., 2020; Wang et al., 2021). Classic single-cell isolation methods include micromanipulation, fluorescence-activated cell sorting (FACS), and laser capture microdissection (LCM), all of which are well-established and widely used (Tan et al., 2010; Hodne and Weltzien, 2015; Hu et al., 2016). Micromanipulation and LCM allow a visualized analysis of cells, capturing high-resolution images of each cell for study (Hodzic, 2016), but the throughput is small, the number of cells that can be analyzed is limited, the operation is complex, and the risk of contamination is high. FACS, in comparison, can process thousands of cells quickly and perform subsequent analysis combined with flow cytometry, but it cannot give detailed morphological information. Compared with traditional screening techniques, droplets can encapsulate cells and the molecules secreted by cells at the same time (Baron et al., 2019; Buhari et al., 2020; Ludwik et al., 2021). Due to the small volume of droplets, molecules secreted by encapsulated cells quickly reach detectable concentrations (Baret et al., 2009; Agresti et al., 2010; Kintses et al., 2012), and the encapsulated cells can be lysed to determine intracellular molecules (Najah et al., 2012). Because the released DNA or RNA is amplified in droplets, rapid genetic analysis of cell biochemistry is possible (Beer et al., 2008; Schaerli et al., 2009; Hatch et al., 2011). Thus, droplet microfluidics can be combined with FACS to achieve efficient and high-throughput screening. Additionally, droplet microfluidics is not limited to detecting cell surface markers, It can also quickly identify and isolate single cells or 3D cell cultures (Anagnostidis et al., 2020), and the analysis is highly flexible.

**FIGURE 5:**
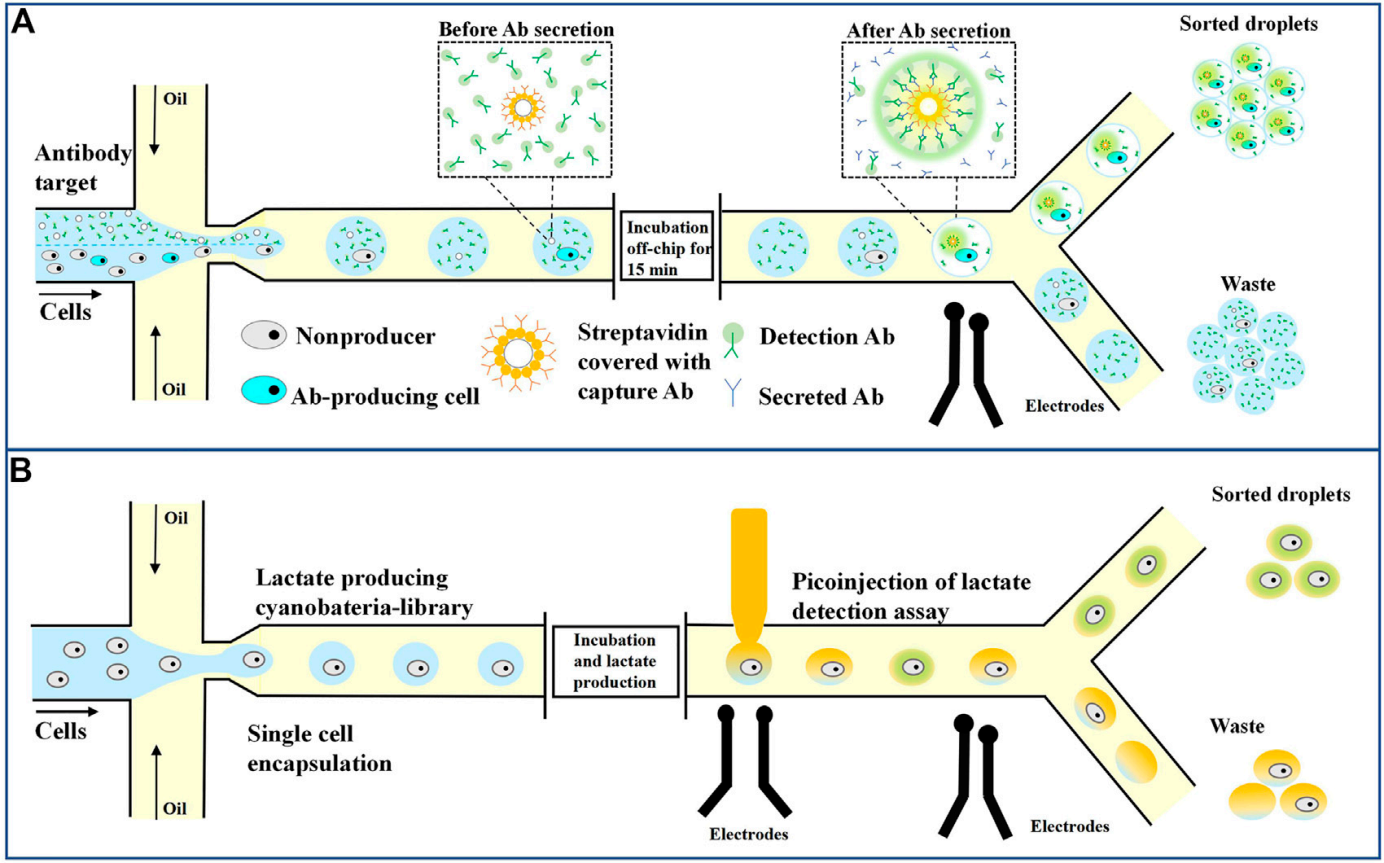
Droplet microfluidics for single-cell screening. **(A)** Sorting of antibody-secreting cells. Secreted antibodies were captured on the bead with fluorescently labeled detection antibodies, leading to the localization of fluorescent signals on the bead, and fluorescent droplets can then be sorted (Mazutis et al., 2013). **(B)** Lactate-producing cyanobacteria are encapsulated as single cells in 10 pL droplets. Following lactate production through incubation, an assay is performed where the droplets are injected with an enzyme that catalyzes the activation of a fluorescent dye in the presence of lactate. The fluorescent droplets are analyzed and sorted based on the strength of the signal (Hammar et al., 2015).

The first single-cell microfluidic screening platform captured single cells for analysis based on droplet capture arrays. The Quake group designed a complex microfluidic chip consisting of multiple valves for the first time, where the valves could be closed to create micrometer scale spaces separating individual cells (Unger et al., 2000). Although the chip has been commercialized and used to effectively screen individual cells that produce antigen-specific antibodies (Love et al., 2006), the maximum number of single cells that can be analyzed is only 4,104, which limits the throughput of the experiment, and the system thus cannot be applied to high-throughput cell screening. In contrast, droplet-based microfluidic platforms can simultaneously process millions of droplets (cells) to achieve high-speed and high-throughput cell sorting (Debs et al., 2012). Mazut developed a droplet microfluid-based platform for high-throughput screening of single antibody-secreting cells from many non-secreting cells (Mazutis et al., 2013). Figure 5A shows 9E10 cells (mouse hybridoma cells that secrete IgG antibodies against human C-MYC protein), and K562 cells (human chronic myeloid leukemia cells that do not produce antibodies) were used, where the latter was used as a negative control (Mazutis et al., 2013). A droplet was encapsulated with a tested cell, a green fluorescent labeled goat detection antibody, and an affinity bead coated with goat anti-murine Fc capture antibody. In the sandwich test, antibodies secreted by the cells are captured on the surface of the beads and detected with a fluorescently labeled secondary antibody. The fluorophore concentrates on the beads to produce a distinguishable signal. Fluorescence is detected by the microfluidic sorter, and bead-free, cell-free, and antibody-secreting cells flow into one channel, while droplets containing antibody-producing cells and beads are collected in the other channel. This method has a fast-screening speed, and a single cell in the 50 pL droplet secretes enough antibodies to be detected within 15 min. The high viability of the cells in the droplet allows the screening of primary cells isolated from human blood without the need for cell immortality.

The fluorescence-based binding assay described also applies to detecting secretory molecules, such as cytokines and growth factors, in the presence of fluorescently labeled ligands (Sesen and Whyte, 2020). In addition, the method can also be adapted for high-throughput enzyme screening and directed evolution or for screening antibodies and other molecules that inhibit enzyme activity by using fluorescent substrates or other fluorescence analysis (Reymond et al., 2009; Abrishamkar et al., 2022; Yuan et al., 2022). Colin et al. (2015) used a microdroplet system to screen out new hydrolases from macroscopic genomic sources. First, they induced the expression of a metagenomic library containing more than a million variants from different environmental sources in *E. coli* cells. Then, they encapsulated these cells in microdroplets with a lysis buffer and a fluorescent substrate. After cell lysis, high-intensity fluorescent droplets were collected by fluorescence-activated droplet sorting (FADS), and more than 20 million droplets were screened within 2 h. The metagenomic screening system identified six unique sulfatase variants and eight unique phosphotriesterases. Droplet microfluidics can also screen genetically engineered bacteria, such as genetically modified cyanobacteria and wild-type ethanol-producing and lactic acid-producing strains. Abalde and Hammar’s team cultured the cells in droplets and made the bacteria secrete ethanol or lactic acid (Abalde-Cela et al., 2015; Hammar et al., 2015). The droplets were then fed into a microfluidic device with electrocoalescence to fuse the solution containing the detected enzyme with the droplets, as shown in Figure 5B (Hammar et al., 2015). After incubation, and enzymatic reactions activated the fluorescent substrate in the presence of the desired metabolites. Finally, fluorescent signal sorted and collected bacteria containing high levels of metabolic substrates.

### 3.3 Single-cell detection

#### 3.3.1 Droplet microfluidics-based detection

Analysis and detection technology are very important in biological research and development. Data used to characterize the physical properties of droplets, such as size, shape, flow rate, and surface tension, and chemical data, such as the nature and quantity of encapsulated analytes, provide a sufficient experimental basis for quantifying research results. Droplet microfluidics-based detection stands out among other detection techniques because of its multiple advantages. The use of ultrasmall droplet volumes, which makes single-cell and single-molecule analysis possible, facilitates high-rate and large-scale droplet generation conducive to realizing high-throughput single-cell detection. Droplets also allow a variety of flexible operations, such as merging, splitting, and sorting, which can be combined with different operations for multifunctional detection experiments. The excellent specific surface area of droplets enables them to have high mass and heat transfer characteristics, which enables the reactions in the droplets to occur quickly and allow rapid detection (Zhu and Fang, 2013; Liu and Zhu, 2020). Commonly used droplet detection techniques include optical, electrical, and mass spectrometry. The most widely used optical detection technology is fluorescence detection, a method to obtain target analyte information from droplets through endogenous fluorescent molecules or fluorescence markers. Fluorescence microscopy allows the quantification of concentrations and differentiation of droplet expression, and ultrahigh-throughput droplet detection can be achieved by integrating high-speed cameras and fluorescence imaging technology. To further improve the throughput of fluorescence imaging, the most effective method is to expand the field of view to increase the number of droplets in a single image; however, the sensitivity of detection is reduced due to light dispersion induced by a large field of view. Lim et al. (2013) designed a microfluidic micro-optical lens array to obtain better sensitivity in a large field of view. By combining the micro-optical lens with the mirror structure on the chip channel wall, the fluorescence signal could be enhanced by approximately 8 times. Kim et al. (2015) also increased monitoring throughput by integrating sensor arrays into the microfluidic chip. A two-dimensional CMOS sensor array was fabricated at the bottom of 16 microchannels, which could detect droplets in parallel without affecting the collection efficiency of fluorescent droplets. An ultrahigh detection throughput of 25.4 kHz could be achieved with this device.

#### 3.3.2 Single cell detection

**FIGURE 6:**
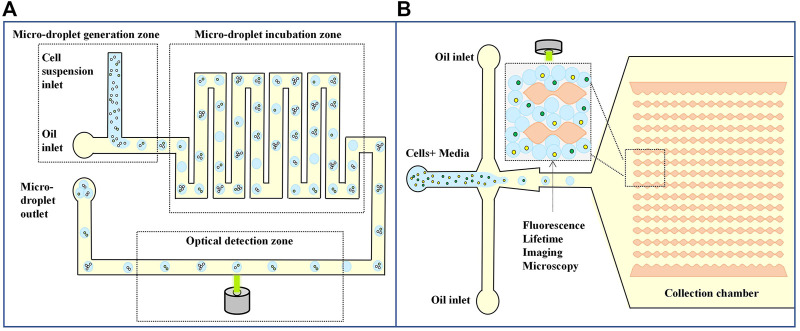
Droplet microfluidics for Single cell detection. **(A)** Schematic diagram of a microfluid based optical sensing device for labeling free detection of circulating tumor cells (Chiu et al., 2015). **(B)** Label‐free metabolic classification of single cells in droplets using the phasor approach to fluorescence lifetime imaging microscopy (Ma et al., 2019).

Fluorescence imaging combined with droplet detection is highly sensitive and has been widely used in high-throughput screening and other single-cell analysis studies requiring delayed droplet monitoring. Chiu et al. (2015) developed a method for quantifying the number of tumor cells from background leukocytes based on analyzing metabolites, which may be applied to counting circulating tumor cells from whole blood. Tumor cells have strong metabolic lactic acid production, so tumor cells can be distinguished from normal cells by measuring the level of lactate secretion. In the droplet system, secreted lactic acid is captured in a small volume, facilitating the highly sensitive detection of individual tumor cells with fluorescent lactic acid reagents. As shown in Figure 6A; Chiu et al. (2015) designed a microfluidic-based optical sensing device for label-free detection of circulating tumor cells. Briefly, the microfluidic device consists of micro-droplet generation, micro-droplet incubation, and optical detection zones. In the micro-droplet generation zone, a cell suspension supplemented with a fluorescence-based lactate reagent was continuously delivered to the micro-channel for oil flow through a micro-capillary tube. By this simple process, cell suspension micro-droplets were generated in a continuous manner. The droplets were subsequently delivered to the droplet incubation zone. When the zone was fully filled with droplets, the input flows were stopped. The droplets were incubated for 3 h to cause the cancer cells in the droplets to produce lactic acid for measurement, and this process allows the development of fluorescence for the subsequent optical sensing step. After a static incubation, the micro-droplets were delivered to the micro-channel parallel to the micro-droplet generation, and incubation zones for the quantification of lactic acid through the fluorescence-based optical sensing. They showed that the method could detect 10 tumor cells from 10 mL of white blood cells.

Another metabolism-based single-cell assay measures nicotinamide adenine dinucleotide (NADH) changes in cellular metabolites. Due to increased glycolysis, tumor cells had a higher free/bound NADH ratio than normal cells. Ma et al. (2019) combined droplets with fluorescence imaging methods, encapsulated single cells in droplets, and measured NADH metabolism changes to distinguish different leukemia cell types and stages of the cell cycle. The microfluidic device has an expansion flow focusing geometry as shown in Figure 6B. The device consists of two inlets: Cells suspended in the culture media is injected through the left inlet while the oil phase enters the device through the other inlet. Two different types of human leukemia cells K562 erythromyeloid and Jurkat T-cell leukemia were encapsulated one-to-one in the droplets. The droplets are then delivered to the next region, which is used for single cell fluorescence lifetime imaging microscopy characterization. The droplets if collected in the uniquely designed collection chamber. It prevents the droplet motion during fluorescence lifetime imaging microscopy measurements to a considerable extent. This assay uses the droplet microfluidic technology together with the phasor approach to fluorescence lifetime imaging microscopy to study cell heterogeneity within and among the leukemia cell lines. They have extended these techniques to characterize metabolic differences between proliferating and quiescent cells—a critical step toward label-free single cancer cell dormancy research. The result suggests a droplet-based non-invasive and label-free method to distinguish individual cells based on their metabolic states, which could be used as an upstream phenotypic platform to correlate with genomic statistics.

#### 3.3.3 Single cell genomics analysis

**FIGURE 7:**
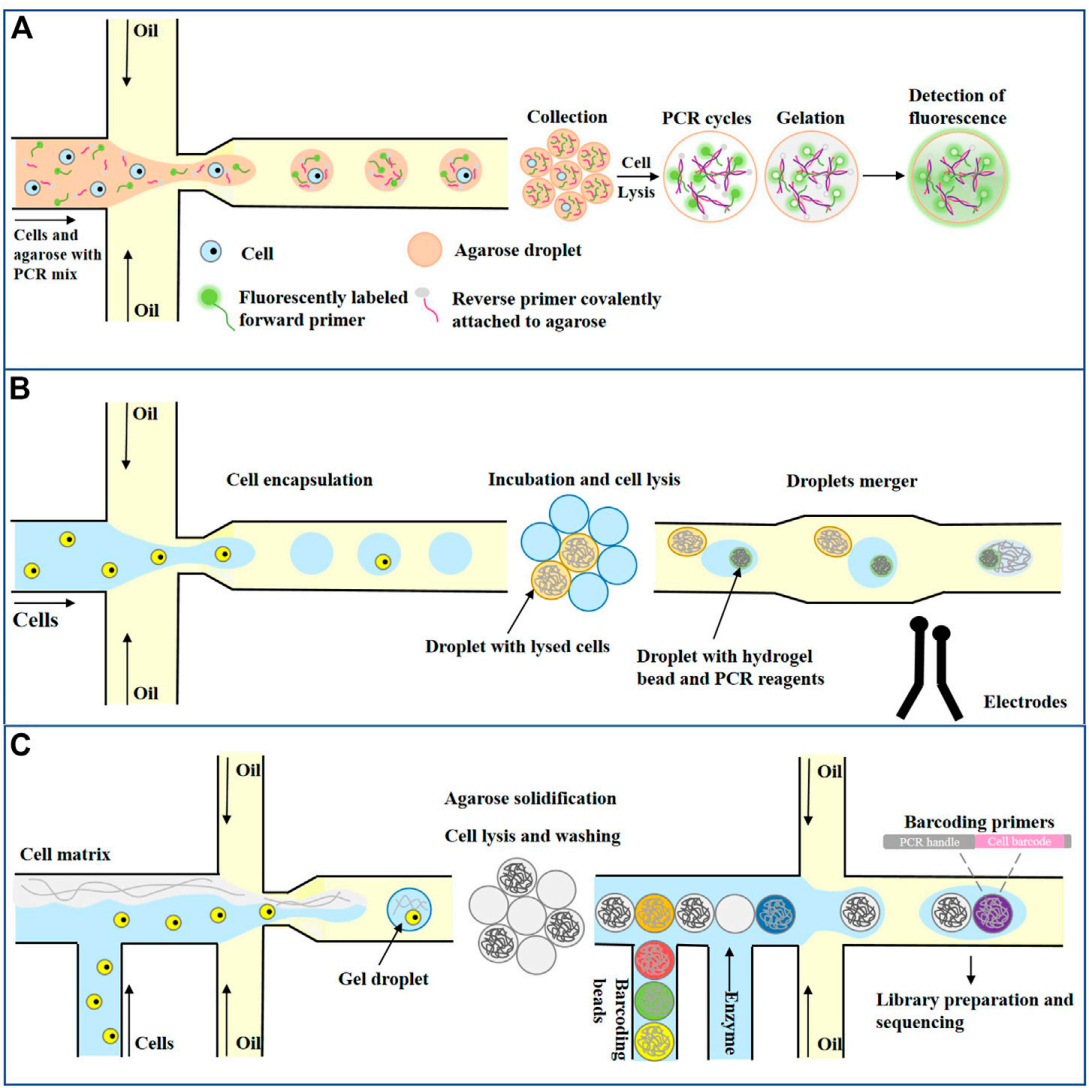
Droplet microfluidics for Single cell genomics analysis. **(A)** Single-cell PCR for emulsions based on agarose gel droplets (Zhu et al., 2012). **(B)** Single lactate-producing cyanobacterial cells are encapsulated in 10 pL droplets. Following lactate production through incubation, an assay is performed where the droplets are injected with an enzyme that catalyzes the activation of a fluorescent dye in the presence of lactate. The droplets are classified according to the fluorescence signal (Pellegrino et al., 2018). **(C)** Single Cell Copy Number Variation (Andor et al., 2018).

Another important application of single-cell assays is targeted and genome-wide analysis at the single-cell level and quantifying single-cell protein levels (Lareau et al., 2019; Bounab et al., 2020; Matua et al., 2020). Genomic analysis of individual cells provides a unique opportunity to study the genomes of rare or unculturable microorganisms, tumor cell heterogeneity, or drug resistance acquired during treatment (Liu et al., 2021; Zhou et al., 2021). A key step in single-cell genome analysis is DNA amplification on the scale of picograms or nanograms into micrograms. There are two main amplification strategies. One is targeted genome amplification, which amplifies only targeted fragments using standard PCR cycles in which primers are designed to bind only to specific sequences of interest in the genome. The other is whole-genome amplification (WGA), in which primers must bind to multiple places in the genome to amplify the complete DNA strand. Zhu et al. developed an emulsion PCR method using agarose as a continuous phase to amplify single-cell-derived DNA molecules (Zhu et al., 2012). As shown in Figure 7A, each agarose droplet prepared by cross-focusing flow contained 0.5–2 cells and a PCR mixture with forward primers and enzymes containing fluorescence markers. Reverse primers were covalently attached to agarose, and further PCR was performed to amplify the target DNA of interest after cell lysis. The agarose used in the experiment is a low gelation agarose. Once melted, it remains liquid at room temperature, which is conducive to the generation of droplets. After freezing at 4°C, agarose solidified, which is conducive to subsequent detection. After amplification, Agarose was solidified into solid beads, and a fluorescently labeled PCR amplicon was attached to the agarose matrix. Finally, the unused fluorescently labeled forward primers were washed away, and the samples were analyzed using fluorescence microscopy or flow cytometry. Because the primers are fixed in the droplet, the amplification efficiency of the target molecule is greatly improved, and the experiment time and cost are reduced. Agarose droplet microfluidic ePCR shows one order of magnitude increase in the efficiency of cells and primers co-encapsulation since primers are part of the agarose liquid phase, and are therefore present in all droplets. Increase in co-encapsulation efficiency enables characterization of whole-cell populations, and decreases the duration and costs of the experiment.

Previously, single cell DNA analysis was most commonly performed with laboratory-developed approaches relying upon FACS sorting to first isolate single-cells in 96- or 384-well plates. Not only are these approaches laborious and slow, but they also utilize significant amounts of reagent to generate sequence information. The droplet microfluidic method can generate sequence libraries in a short time and, through the use of picoliter volume droplets, consumes minimal reagent to barcode genomic DNA. These key features significantly lower the barriers for performing single-cell DNA sequencing and promise to make high-resolution analysis of clonal architecture within tumors routine. Mission Bio (Pellegrino et al., 2018) developed a single-cell genome analysis platform that has been put into commercial application. As shown in Figure 7B, a single cell and protease were first encapsulated in the droplet, and the temperature was raised after cell lysis to inactivate the protease. Subsequently, each droplet containing the single-cell genome was fused with a second droplet containing PCR reagent and barcode hydrogel beads (1:1 ratio) by electropolymerization. Barcode hydrogel beads were labeled with oligonucleotides containing cell-specific barcodes and gene-specific primer sequences. After the hydrogel beads were encapsulated with the genome-containing droplet contents, primers labeled with hydrogel beads were released by UV irradiation, and PCR amplified the target DNA sequence to encode the cell barcode. Finally, the oil phase was removed, and the droplets were collected to prepare the library for sequencing. Pellegrino et al. (2018) used this approach to demonstrate a proof-of-concept experiment using 62 DNA targets to analyze the genetic heterogeneity of a single AML cell. As a demonstration of the technology, they characterize longitudinal samples from AML patients and uncover features of clonal architecture that are not available from bulk sequencing data. This rapid, cost-effective, and scalable approach promises to make routine analysis of genetic variation in tumors a reality.

Droplet microfluidic not only has broad application prospect in single cell targeted genome analysis, but also plays an important role in whole genome analysis. Single-cell DNA sequencing (scDNA-Seq) can identify somatic genetic changes, such as somatic cell copy number variation (CNVs). For cancer, single-cell CNVs characterize high resolution intratumoral heterogeneity and subclonal structures present in primary tumors, metastases, patient-derived xenografts, and even cancer cell lines. However, the cellular throughput of scDNA-Seq has been limited, with a typical maximum of hundred cells. Greater sampling of tumor tissues provides an opportunity to expand the scope of intratumoral characterization. As a solution that enables massive scale scDNA-Seq, Andor et al. (2018) developed a droplet-based partitioning technology that rapidly processes thousands of cells per sample for library preparation in a highly automated fashion. Using this new approach, they conducted single cell WGS on thousands of cells for nine gastric cancer cell lines and a primary gastric tumor. As shown in Figure 7C, First, single cells are encapsulated at limiting dilution in a gel matrix with paramagnetic particles within a single-use microfluidic chip. Once droplets gelate, cells are trapped inside the beads and are subjected to lysis followed by the removal of all nuclear proteins, while the genomic DNA remains trapped in the gel matrix. Subsequently, purified genomic DNA inside the cell beads is coencapsulated in a second microfluidic device with barcoding beads (10X Barcoded Gel Beads) and enzymes. Importantly, both the cell beads and the barcoding beads are closely packed, allowing high-efficiency co-encapsulation of one cell bead and one barcoding bead in each droplet. DNA inside the droplet is amplified to generate single-cell barcoded libraries ready for sequencing and analysis. Overall, the throughput of this microfluidic based cellular isolation system demonstrated a scale up to tens of thousands of cells per a microfluidic chip. This processing capacity exceeded flow cytometry-based isolation by several orders of magnitude.

## 4 Tumor single-cell immunoassay based on droplet microfluidics

The human body’s immune system is a large and complex reaction system that can cope with and recognize many environmental factors and respond to various signals (Dantzer, 2018; Daëron, 2022). Different types of cells, antibodies, and cytokines interact synergistically to produce immune responses. The dynamic migration of cells and tissues and the dynamic intercellular interactions enhance the complexity of immune system responses (Rivera et al., 2016; Prata et al., 2018). Not all cells fight all pathogens and tumors in the same way. For example, whether cells are alone or in a population. Experiments performed at the population level average the behavior of individual cells and mask differences between individual cell phenotypes, gene expression, protein expression, metabolites, and cell communication (Jammes and Maerkl, 2020; Grigorev et al., 2023). The emergence of single-cell techniques has made it possible to study the immune behavior of single cells. For example, single-cell analysis can study the maturation, activation, and signaling pathways of individual immune cells triggered by various environmental factors (Zaretsky et al., 2012; Junkin and Tay, 2014), as well as the interaction between different immune cells, and it can even help to discover and identify new immune cell subsets (De Rosa et al., 2001).

Single-cell technology involves isolating individual cells from a population to extract data from each cell and obtain cell-related information for analysis. After isolating a single immune cell, multiple experimental operations of DNA and RNA sequencing and protein expression profiles can be performed to map the immune cell lineage and identify immune cell subsets (Kimmerling et al., 2016; Papalexi and Satija, 2018). Rutkauskaite et al. demonstrated the importance of paracrine communication in generating an immune response using single-cell analysis (Rutkauskaite et al., 2022). Gaublomme et al. (2015) introduced a paradigm shift in the field of CD4^+^ T helper cells based on a single-cell transcriptome; they were able to further identify multiple T helper cell subpopulations with different functions to the two identified subpopulations, Th1 and Th2.

In immunology research, flow cytometry is a recognized single-cell analysis tool that can perform high-throughput analysis of thousands of single cells in a reasonable time frame and simultaneously measure multiple parameters (Bendall et al., 2012; Dhoble et al., 2016). However, flow cytometry’s major disadvantages are spectral overlap and the limited applications of isotope-labeled antibodies. Furthermore, flow cytometry is an endpoint measurement tool that can only provide information on immune cell heterogeneity by quantifying static markers on cells. Droplet microfluidics is an emerging and popular technology for studying single cells in recent years. The technology helps scientists identify cellular signaling pathways, map different immune cell subpopulations, quantify secreted molecules, and describe immune responses under different conditions (Kirschbaum et al., 2009; Kellogg et al., 2015; Welsh et al., 2023). Cell culture in droplet microfluidics chips is very effective for distinguishing the heterogeneity of tumor cells and studying the self-organizing characteristics of stem cells and the antagonism between cells (Sinha et al., 2018; Hsieh et al., 2022). These studies on immune cells inspire innovation in immunotherapy methods and help to reduce cytotoxicity and side effects of related therapies (Hemmer et al., 2006; Li et al., 2016).

Microfluidic devices have also isolated circulating tumor cells from clinical samples. Circulating tumor cells contain all tumor-related genes. Knowledge of genetic changes in circulating tumor cells can help select appropriate therapies for different patients, which can be used for diagnosis, prognosis, and creation of patient-derived tumor models (Ruan et al., 2021; Qiu et al., 2022a). High-throughput and parallel microfluidic chips have advantages in screening and developing cancer drugs. Since droplet encapsulation can be targeted to separate and distinguish cells, microfluidic platforms can be used to observe the response of cells to treatment in a short time and develop and screen cancer drugs with high throughput and low cost (Nazari et al., 2021; Petreus et al., 2021). Microfluid-based models have contributed to the development of new therapies, the discovery of new drugs, and the testing of the clinical efficacy of new therapies (Del Ben et al., 2016; Lanz et al., 2017; Qiu et al., 2022b). Sarkar et al. (2015) demonstrated an array-based droplet device that allows longitudinal detection of T-cell activation responses in nanoliter-sized droplets. Their results showed that individual T cells were activated more quickly when they came into contact with dendritic cells than other activation methods. In addition, the authors developed a method to explore the heterogeneity of NK-cell potential lytic behavior (Sarkar et al., 2017). They demonstrated a killing efficiency of 100% of NK cells, in sharp contrast to earlier studies (Dura et al., 2016; Guldevall et al., 2016). To quantify the molecules secreted by cells, Konry et al. (2011) paired cells with functionalized beads or sensing molecules prior to analysis and captured target analytes during cell incubation. Trapping in droplets ensures that encapsulated cells are protected from external factors that may influence their secretory behavior. Due to the secreted molecules being confined to the small volume of the droplets, detection sensitivity is improved.

**FIGURE 8:**
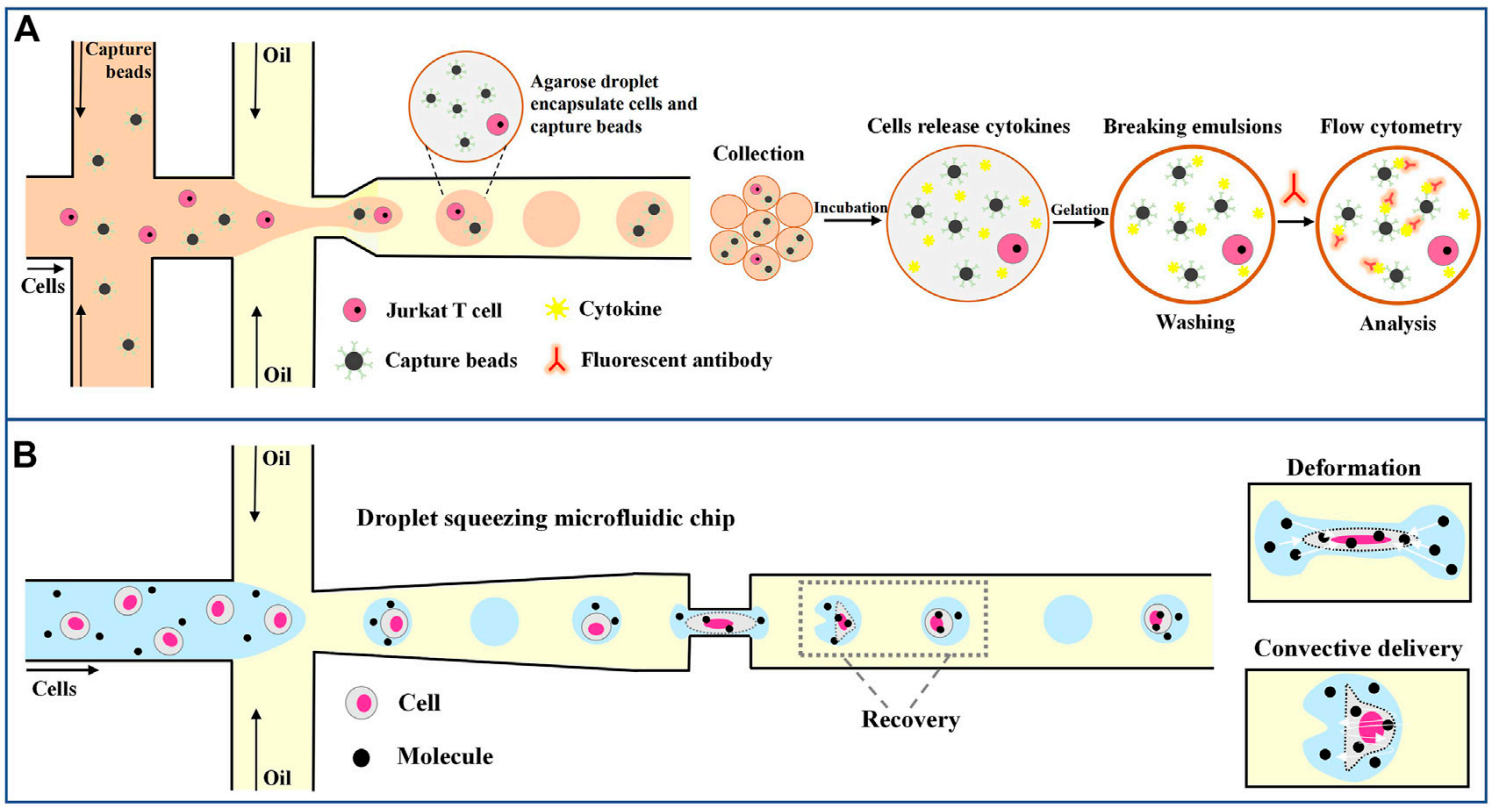
Droplet microfluidics for single-tumor-cell immunoassay. **(A)** Schematic overview of the workflow of the droplet-based microfluidic method for single-cell encapsulation and detection of secreted cytokines (Chokkalingam et al., 2013). **(B)** Molecules and cells are encapsulated in the droplet, and efficient transfection is achieved through deformation recovery when passing through a narrow microfluidic channel (Joo et al., 2021).

Gel droplets also have many advantages over water-based droplets in single-cell analysis. Huck’s lab used agarose droplets to encapsulate Jurkat T cells (Chokkalingam et al., 2013). As shown in Figure 8A, Individual jurkat T cells were encapsulated in agarose gel droplets together with functionalized capture beads. The droplets were collected and incubated at 37°C for 18–24 h. During incubation, individual activated jurkat T cells secrete cytokines that bind to functionalized capture beads confined to the same droplet. After incubation, the droplets were frozen at 4°C to form gel beads, which remained stable and solid at room temperature. After the oil phase was removed, the beads were incubated with fluorescence-labeled antibodies against IL-2, TNF-α and IFN-γ, and the staining intensity of the beads was measured by flow cytometry. This method allows high-throughput detection of cellular heterogeneity and maps subsets within cell populations with specific functions.

In addition, droplet microfluidics can also facilitate the manipulation of cells by manipulating droplets. Joo et al. (2021) invented a microfluidic channel to transfect immune cells through droplets. As shown in Figure 8B, a droplet encapsulates a cell and extracellular material, such as messenger RNA and plasmid DNA. The droplets, and therefore the cells, are squeezed as they pass through the narrow passageway, leading to an increase in transient cell membrane pore size on the cell membrane and the instantaneous transfer of macromolecular substances within the droplet into the cell. After passing this channel, the droplets and cells quickly return to their original shape. By using this method, nearly 98% efficient transfection is achieved, allowing different macromolecules to be delivered into various immune cells, including human primary T lymphocytes, and advancing the field of cancer immunotherapy.

In the past decade, microfluidics technology has greatly improved our understanding of immunology and the study of tumor cells. The technology also provides a unique opportunity to develop new therapeutics and oncology drugs. The introduction of microfluidics made it possible to study T-cell activation at the single-cell level and even brought new insights into the pairing of individual immune cells and cancer cells (Segaliny et al., 2018). Based on this, many scientists have studied the molecular background of cancer immune checkpoints and made discoveries related to cancer immune surveillance (Wuethrich et al., 2019; Strati et al., 2021). In addition, microfluidics will eventually allow the screening of highly specific therapeutic antibodies from human B cells (in cancer patients and disease survivors) (Broketa and Bruhns, 2022; Sun et al., 2022). As antibodies of human origin, these antibodies should be free of any adverse reactions and side effects, and due to their high specificity, the therapeutic effect should be significant. Microfluidic antibody screening can also be used to identify vaccine candidates. Gérard et al. proposed a high-throughput, single-cell screening of IgG-secreting primary cells in combination with a microfluidic droplet system that generated 77 recombinant antibodies from the identified sequences and recovered approximately 450–900 IgG sequences from human memory B cells. The authors further demonstrated the method’s versatility when activated *in vivo* (Gérard et al., 2020). In addition, the systematic sequencing of gradually developed immune sequences will provide further insight into autoimmune diseases. Microfluidics will contribute significantly to future biomedical discoveries.

## 5 Summary and outlook

Droplet microfluidic technology has developed rapidly in the past decade and has solved many problems in biological research. In particular, single-cell technology is the most striking. As shown in Table 1, we list the single-cell technologies described in this article and compare their advantages and disadvantages. Obviously, the single cell analysis technique based on droplet microfluidic has its unique advantages. It provides each cell with a volume nearly equal to the volume of the cell itself while allowing the encapsulation and rapid manipulation of large numbers of cells. Droplet microfluidic chips can facilitate the culture of individually isolated cells and specific cell populations, the targeted screening of secretory molecules such as enzymes and antibodies, rare cell identification, and cell secretion detection. These methods help researchers reveal the heterogeneity of seemingly identical cells in terms of morphology, function, composition, and genetic properties. Droplet microfluidics can also achieve effective and sensitive quantitative detection for small amounts of biomolecules, such as proteins, DNA, RNA, or other cell surface molecules. These developments are invaluable for biotechnology, as the overall analysis of individual cell performance, intracellular processes, and interactions with environmental conditions provide the basis for constructing the entire microbial map. In particular, these methods play an important role in understanding the human immune system by allowing complex immunological problems to be solved that traditional bulk analysis methods cannot solve, such as identifying heterogeneous immune cell behavior, discovering new immune cell subpopulations, and understanding how individual immune cells drive population responses. The droplet-based single-cell analysis contributes to developing new diagnostic tools and personalized drugs for treating cancer, immunosuppressive diseases, and autoimmune diseases (Armbrecht et al., 2017; Golumbeanu et al., 2018). In addition, for vaccine development, it is critical to understand how specific antigens induce effective immunity, and new vaccines with higher clinical efficacy can be developed using antibody screening and quantitative results at the single-cell level (Shembekar et al., 2018). A detailed understanding of cell-cell or cell-pathogen interactions will revolutionize cell biology, especially immunology and cellular immunotherapy.

**TABLE 1:** Comparison of advantages and disadvantages of single-cell assay.

Single-cell technique	Methods	Advantages	Disadvantages	References
Single-cell Culture	Vitro cell culture	Controllable culture condition; Easy to observe; Easy to obtain homogeneous cell population; Low cost	Single cell heterogeneity could not be characterized	Eyer et al. (2017), Eibl et al. (2018), Jensen and Teng (2020), Badr-Eldin et al. (2022)
	Droplet single-cell culture	Low reagent consumption; Reflect the heterogeneity between individual cells; High throughput	Cell vitality is slightly decreased; Complicated experimental operation	Huebner et al. (2007), Köster et al. (2008), Nakagawa et al. (2021), Pang et al. (2021), Seiler et al. (2022)
	Hydrogel droplet culture	Natural biocompatibility; Phase transformation in response to external physical and chemical changes; High throughput	Uneven hydrogel formation; High cytotoxicity	Huang et al. (2006), Tan and Takeuchi (2007), Zhang et al. (2007), Leng et al. (2010), Moyer et al. (2010), Dolega et al. (2015), Utech et al. (2015), Choi et al. (2016), Shao et al. (2020), Lin et al. (2021), Tiemeijer et al. (2021)
Single-cell Screening	LCM	Allow a visualized analysis of cells; Capability of capturing high-resolution images	Low throughput; Complicated operation; High contamination risk	Tan et al. (2010), Hodne and Weltzien (2015), Hodzic, 2016; Hu et al. (2016)
	FACS	High throughput; Rapid analysis speed	Cannot give detailed morphological information	Baron et al. (2019), Buhari et al. (2020), Ludwik et al. (2021)
	Single cell screening based on droplet microfluidics	High sensitivity; Capability for multiple element analysis; Rapid analysis speed; High throughput	Requiring sample pretreatment; High cost	Unger et al. (2000), Love et al. (2006), Beer et al. (2008), Baret et al. (2009), Reymond et al. (2009), Schaerli et al. (2009), Hatch et al. (2011), Debs et al. (2012), Najah et al. (2012), Mazutis et al. (2013), Abalde-Cela et al. (2015), Colin et al. (2015), Hammar et al. (2015), Anagnostidis et al. (2020), Sesen and Whyte (2020), Abrishamkar et al. (2022), Yuan et al. (2022)
Single-cell Detection	Fluorescence imaging combined with droplet detection	High sensitivity; High throughput; *In situ* analysis; Readily available	Mostly need fluorescence labeling	(Chiu et al., 2015)- (Ma et al., 2019)
	Single cell genomics analysis based on droplet microfluidics	Capability of generating sequence libraries in a short time; Less reagent consumption	The high cost of instruments cannot be extended to clinical applications	Zhu et al. (2012), Andor et al. (2018), Pellegrino et al. (2018), Lareau et al. (2019), Bounab et al. (2020), Matua et al. (2020), Liu et al. (2021), Zhou et al. (2021)

However, there are challenges associated with research on droplet microfluidics and single-cell analysis, and there are still many problems to be overcome. Most current methods are limited to a single type of information output, such as data related to DNA, RNA, or protein. Quantifying multiple layers of information simultaneously at a single cell level is a current goal of scientists in the field. Another major challenge of the single-cell approach is the lack of information on dynamic processes, further complicated by differences in cell heterogeneity in samples. Moreover, tissue separation is required before single-cell encapsulation, which inevitably leads to the loss of spatial information about the original location of cells in the tissue. Research into the spatial transcriptome seeks to combine cellular transcriptome information with spatial organization within tissues using computational techniques to obtain missing information in both cases (Eyer et al., 2017). The combination of electrical detection and droplet microfluidics based on mass spectrometry technology has also made great advances in biological applications and medical diagnostics. Droplet technology has been transferred from laboratory experiments to the clinic, and there is a higher requirement for the droplet environment for cell survival. Considerable efforts are needed to provide an environment that does not interfere with cell characteristics.

Even so, droplet microfluidic diagnostics also have promising applications. The simplicity and cost-effectiveness of microfluidic diagnostics will enable single-cell research to be applied in biomedicine, chemical systems, and clinical biology.
